# Catastrophic expenditures and impoverishment due to out-of-pocket health payments in Kosovo

**DOI:** 10.1186/s12962-018-0111-1

**Published:** 2018-07-28

**Authors:** Fatime Arenliu Qosaj, Guenter Froeschl, Merita Berisha, Bashkim Bellaqa, Rolf Holle

**Affiliations:** 1 Center for International Health at Ludwig-Maximilians-Universität München, Leopoldstr. 7, 80802 Munich, Germany; 2 Division of Infectious Diseases and Tropical Medicine, Medical Center of the University of Munich (LMU), Leopoldstr. 5, 80802 Munich, Germany; 3 National Institute of Public Health of Kosovo, Rr. Mother Theresa p.n., 10000 Prishtina, Kosovo; 4 Medical Faculty Prishtina University, Rr. Bulevardi i Dëshmorëve p.n., 10000 Prishtina, Kosovo; 5 University of Mitrovica, Rr. Parku Industrial p.n., 40000 Mitrovica, Kosovo; 6 Helmholtz Zentrum München, German Research Center for Environmental Health, Ingolstädter Landstr. 1, 85764 Oberschleissheim, Germany

**Keywords:** Out-of-pocket payments, Catastrophic health expenditures, Impoverishment, Kosovo

## Abstract

**Background:**

The current health system reforms in Kosovo aim to improve health status through universal health coverage. Risk pooling and ensuring access to necessary care without financial hardship are envisaged through compulsory health insurance. We measure the level of financial risk protection through two commonly applied concepts: catastrophic health expenditures and impoverishment.

**Methods:**

Data from the 2014 Kosovo Household Budget Survey were used to estimate catastrophic health expenditures as a percentage of household consumption expenditures at different thresholds. Poverty head counts and gaps were estimated before and after out-of-pocket (OOP) health payments.

**Results:**

Approximately 80% of the households in Kosovo incurred OOP health payments. Most of these expenditures were for medicine, pharmaceutical products and medical devices, followed by diagnostic and outpatient services. Hospital services and treatment abroad were less frequent but highly costly. Although households from the upper consumption groups spent more, households from the lower consumption groups spent a greater share of their consumption expenditures on healthcare. The catastrophic health expenditure head count showed an increase, while the impoverishment and poverty gap remained stable compared to 2011. Regression analysis showed that age of the household head, insurance coverage, household size, belonging to the lowest consumption expenditure quintiles, and having disabled and aged household members were significant predictors of the probability of experiencing catastrophic health expenditures.

**Conclusions:**

Ongoing financing reforms should target the lower income quintiles and vulnerable groups, pharmaceutical policies should be revisited, and the internal referral system should be strengthened to overcome excessive spending for treatment abroad.

## Background

Universal health coverage (UHC) means ensuring that everyone, everywhere can obtain quality health services without financial hardship. While UHC is a reality in some countries, it remains a goal in others. To confront this challenge at the global level, the United Nations General Assembly (UNGA) called on all countries to plan for or direct the transition of their health systems toward UHC [1]. In September 2015, the UNGA adopted 17 Sustainable Development Goals (SDGs). In particular, SDG #3 aims to ensure healthy lives and to promote well-being for people of all ages through UHC, which can be monitored by measuring access to essential quality health services and the level of financial risk protection [2].

The level of financial protection from out-of-pocket (OOP) health payments is estimated through two statistical parameters: (i) the proportion of a country’s population that has a high share of OOP spending for healthcare, which is considered a substantial financial burden on household budgets and is defined as catastrophic expenditures; and (ii) the proportion of the population that falls below the poverty line due to OOP health spending, which is defined as impoverishment. Governments, the World Bank (WB), the World Health Organization (WHO) and civil society organizations have recognized that ensuring access to health services for everyone without causing financial hardship is crucial to sustainable economic growth and development. Therefore, closely monitoring the progress toward these goals can support evidence-based policy decisions and enrich the global knowledge regarding the approaches to achieving UHC despite severely limited resources.

At the global level, UHC is considered a sustainable development goal. In the WHO European Region, UHC is considered a central strategy for achieving the goals of Health 2020 [3]. However, the implementation of this strategy through different health systems has been heterogeneous [4, 5]. While many countries in the WHO European Region are considered to have achieved UHC, some countries continue to report catastrophic and impoverishing OOP health payments [3]. In addition, there is increasing evidence at the global level regarding catastrophic expenditures and the impoverishment effects of OOP health payments [6].

Interest in catastrophic and impoverishing health payments as an indicator of the functioning of a healthcare system at the country level has increased over the past decade [5, 7–10]. Despite different survey methodologies, recall periods, levels of disaggregation of health expenditures, and the different levels of financial protection achieved, published evidence continues to show a socioeconomic gradient in OOP health payments and recommends increasing the availability of healthcare services in conjunction with risk protection policies [11, 12].

Country-level studies on OOP health payments have led to comparative regional and global studies on catastrophic health expenditures and their determinants [12–16]. Globally, more than 800 million people spend at least 10% of their household budget to pay for health services, and approximately 100 million become indigent each year because of high OOP health payments [2]. Comparative regional cross-country studies using comparable evidence have found varying results, including the fact that the prevalence of catastrophic health expenditures in countries within a region varies widely [16]. Catastrophic health expenditures occur at all income levels but are most severe in low-income countries [17]. OOP health payments exacerbate the prevalence and depth of poverty, and borrowing and selling assets have been found to be common coping mechanisms to address high health expenditures in some African countries [14, 15]. A comparative study in the western Balkans using living standard measurement surveys and equivalent surveys in the region from 2000 to 2005 indicated that the catastrophic and impoverishment effects of OOP health payments were more severe in Kosovo and Albania than in Bosnia and Herzegovina, Montenegro and Serbia [13]. Since that study, however, there has been a research gap with regard to monitoring the financial burden caused by OOP health payments in Kosovo. In 2014, Kosovo’s government implemented reforms to move the country toward UHC. Thus, monitoring the financial burden caused by health payments has become imperative.

### Health system reform in Kosovo, 1999–2014

The public health system in Kosovo is mainly funded through taxes. Health services are provided at primary, secondary and tertiary levels of care, and essential drugs are provided free of charge [18]. Except for vulnerable population groups that are exempt by law, patients pay user fees. All patients must pay OOP for drugs that are not included on the essential drugs list. The private sector is funded by OOP payments, private insurance and government program for treatment outside public health facilities. Financed mainly by taxes, public health expenditures account for approximately 60% of total health expenditures. OOP health payments represent 38% of total health spending, and such payments increased the poverty head count by 7% in 2011 [19]. The relatively high OOP health spending at the household level in Kosovo is also a result of the limited public health spending as a share of GDP (2.9%) and of total government spending (9%) (2012), both of which are lower than the EU averages of 5.5 and 13%, respectively [19].

Since 1999, the healthcare system in Kosovo has been undergoing permanent reforms. Because it aspires to join the EU, Kosovo is aiming to enact EU healthcare policies and actions, to protect and improve the health of its citizens, to support the modernization of the country’s infrastructure and to improve the efficiency of its health system [20]. In 1999, the WHO initiated a process to define a healthcare policy framework for an emergency period [21]. This policy framework included components of healthcare sector reform that were consistent with normative health policies in much of Eastern and Central Europe, i.e., introducing the family medicine concept to already decentralized primary healthcare services, developing community mental health services, and investing in family medicine specialization and bachelor-level nursing education. The framework also introduced the concepts of patients’ rights and quality of care. These and other initiatives contributed to longer-term reforms and development. In 2000, the policy framework was revised by a larger group of national professionals together with the WHO in a secretarial role. Since then, several strategic healthcare sector documents have been advanced and approved, and the emergency phase was replaced by a development agenda in which financial barriers that exclude people from utilizing public primary healthcare and hospital services, the use of private clinics and people seeking treatment abroad became concerns [18]. In 2011, pharmaceuticals represented 85% of OOP health payments in Kosovo [19]. The primary goals of the recent healthcare financing reform include providing quality healthcare services, achieving UHC, addressing concerns regarding adequate and equitable access to healthcare [22–24]. The Health Law (2012) and the Health Insurance Law (2014) defined the legal framework for establishing the three pillars of the recent reform: chambers of healthcare professionals, Kosovo Hospital University Clinical Services and a mandatory health insurance scheme funded through general taxes and mandatory insurance premiums. Mandatory health insurance premiums as a new source of financing for healthcare were to be used to improve the quality of care and increase the availability of drugs and accessibility to services, especially for the poor [19].

We use data from the 2014 Household Budget Survey (HBS) to assess the level of financial risk protection at the onset of these health reforms in Kosovo. To measure the impact of OOPs on households, we estimate (i) the burden of OOP direct medical expenditures in Kosovo, (ii) the incidence of catastrophic health expenditures, and (iii) the effect of health spending on national poverty estimates. Further, we analyze (iv) the association between catastrophic health expenditures and demographic, socioeconomic and other factors in Kosovo. This study is of special interest for Kosovo because it establishes a baseline and a method for monitoring the impact of the healthcare financing reforms on OOP health payments and health equity. Moreover, it provides useful insights into the determinants of catastrophic health expenditures that can be used to enhance programs targeting the poor. Furthermore, empirical findings from a newly established state add data to the global pattern of the impact of OOP health payments.

## Methodology

### Data

In this cross-sectional study, we used data from a national representative sample of the Kosovo Household Budget Survey (HBS), which is a rotating panel survey. Data were collected from 2375 of initially planned 2400 households from 300 enumeration areas. With 200 households being addressed per month, the data collection lasted from January 1st to December 31st, 2014. The recording periods were evenly distributed over the survey period to even out the effects of monthly, seasonal or other temporal consumption expenditure variations [25].

The households were selected in two stages from the census sample from 2011 and divided into 14 strata by region and by urban versus rural residency. Sampling weights were taken into account throughout the analyses. More details on the sampling can be found in the census report and HBS report by the Kosovo Agency of Statistics (KAS) [26].

For most variables, the household was the basic unit of observation; however, data were also collected for each household member. Through the HBS questionnaire, the KAS collected data on demographic characteristics, consumption expenditures, self-consumption (non-monetary expenditures for food or other goods and services obtained without payment from the household’s own production of goods and services), household income and socioeconomic status. Data were collected through paper-based, face-to-face interviews during three repeated visits over a period of 2 weeks. The sample plan was elaborated by the KAS, taking into account the representation and limitations of response burdens. The response rate for the initial sample was 82%. Replacements were made for 397 households, and the originally planned sample size was nearly reached [26].

### Definition of variables

The 2014 HBS source variables are divided into twelve categories [27]. The following variables were defined and constructed based on the source variables according to the KAS methodology:

*Household* is defined as a group of persons living together in the same dwelling unit who share expenditures for the essentials of living, pool their income and resources and have family and emotional ties.

*Household consumption expenditures* (HCE) are annual payments, both monetary and in kind, for all goods and services, including the monetary value of self-produced goods and services that are consumed. HCE (excluding durable goods and rent) are used to estimate effective income after basic subsistence needs have been met. This estimate is believed to reflect the ability to pay more accurately than the income reported in household surveys, especially in low and middle-income countries, where informal labor employment is more common, income sources tend to change more frequently and more goods are self-produced [6, 12]. In 2013, on average, 37% of the total employed workforce in Kosovo was not legally declared, while over 60% of Kosovo’s population was dependent on labor in agriculture, and 70% of the workforce in that sector was undeclared [28].

*Food consumption expenditures* represent the annual amount spent on food, including in-kind and self-produced food.

We report annual household consumption and non-food consumption expenditures per adult equivalent. Because HCE are associated with household size and demographic characteristics, households were adjusted for the age of their members and the household size due to considerations of economies of scale: as household size increases, consumption does as well, but not proportionally. To allow for comparability of the data, we opted for the equivalence scales used by the KAS, with an assigned value of 1 for adult members of a household and 0.75 for children. This assigned equivalence value for children is higher than the value assigned for children by the Organization for Economic Co-operation and Development (OECD) in its equivalence and modified scales (0.5 and 0.3 per child, respectively), which would yield an overestimation of children’s consumption in Kosovo in this study [29].

*OOP health payments* represent households’ health care expenditures for medicine, pharmaceutical products and medical appliances, public and private outpatient services at the primary level, hospital services, dental services, and diagnostic services performed in specialized diagnostic facilities as well as treatment abroad. These expenditures also include spending on traditional medicine but exclude transportation costs and health insurance premiums.

All values used in the analyses are annual figures. The year 2002 is the base year for the Kosovo consumer price index (CPI). All monetary values are normalized using the 2014 CPI for all items in real prices [30]. The local monetary values were then converted into the GDP PPP conversion rate of 1 Kosovo Euro = 0.327 PPP-adjusted Euros [31].

We report the annual absolute poverty line per adult equivalent as estimated by the KAS. The poverty line is estimated using the cost of basic needs method. *Absolute poverty* represents the updated (2002) poverty line over time (2014) to account for changes in prices so that it reflects the same set of basic food and non-food needs. The poverty line represents the sum of food and non-food components. The food component represents the cost of a calorie intake of 2100 kilocalories per person per day, and the non-food component includes the cost of other essentials for clothing and shelter, excluding health care [32].

### Construction of statistical parameters

Using the World Bank (WB) methodology presented by O’Donnell et al. in 2008, we assess financial risk protection by estimating OOP health payments, and we present the consequences as (i) catastrophic health expenditures once they exceed a certain share of the household’s expenditures; (ii) impoverishing health expenditures once they are high enough to push a household below the poverty line and increase the poverty head count; and (iii) the poverty gap, which is the amount needed to push a household up to the poverty line and financially protect it from the consequences of its OOP health payments [6].

C*atastrophic health expenditures* are defined in this study as 10% of total household consumption and 40% of non-food household consumption. In the literature, when total household expenditures are used as the denominator, 10% is the most common threshold to define catastrophic payments [6]. This is considered an approximate threshold beyond which a household is assumed to have to choose between healthcare and other basic needs. In contrast, WHO researchers have used “capacity to pay” (non-food expenditures) as the denominator, with 40% set as the threshold [33]. For comparison, we also present different thresholds of OOP health payments from 5–25% when using HCE as the denominator and 15–40% when using non-food household consumption in Eqs. 18.1–18.3 [6]. The choice of threshold is a matter of judgment, and we leave it to the reader to determine the propriety. In the multivariable logistic regression model for catastrophic expenditures, we use the 10% level of HCE as the threshold.

The incidence of catastrophic health expenditures is defined by the *head count ratio* of the percentage of households whose OOP health payments exceed the above-defined threshold in a given time period.

The intensity of catastrophic health expenditures is measured through the following variables:

*Overshot* is the depth of catastrophic payments. Overshot is defined as the average percentage of OOP health payments that exceed the threshold, which in this study is 10% for HCE or 40% for non-food expenditures across the entire sample.

*Mean positive overshot* is the average percentage of OOP health payments that exceed the threshold of 10% of HCE or 40% of non-food expenditures across households that exceed either threshold, respectively.

Household annual consumption poverty at the population level is measured through the following variables:

The *pre*-*payment head count* measures the percentage of individuals whose consumption per adult equivalent is less than the estimated poverty line for 2014 before spending for OOP health payments over the entire reference population. This percentage equals the *poverty head count*.

The *post*-*payment head count* measures the percentage of individuals whose consumption per adult equivalent is less than the estimated poverty line for 2014 after accounting for OOP health payments over the entire population.

The *poverty gap* is defined by the average difference between poor households’ expenditures and the poverty line before and after OOP health payments. The poverty gap measure indicates how much would be needed in terms of monetary funds to transfer to the poor to bring their expenditures up to the poverty line. The impoverishment measures are calculated at the population level.

A logistic regression model was built to identify the variables associated with catastrophic health expenditures. *Catastrophic health expenditures* (*dependent variable*) are considered to have occurred at the household level if OOP health payments are equal to or more than 10% of the HCE. Based on the existing literature, and reasonably conceptualized to the Kosovo context with very limited social assistance programs, for the independent variables, we considered categorizations of the sociodemographic characteristics of households that are potentially related to catastrophic health expenditures [9–11]. Table 1 outlines and describes the independent variables analyzed for their association with catastrophic health expenditures.

**Table 1:** Outline of the variables used in the regression analysis

Independent variable	Description	Measurement
Household size	Total number of people living in the household	Continuous variable
Education	Level of education of the head of the household	Dummy variable:
		0 = primary or lower
		1 = secondary or higher
Most vulnerable age groups	At least one or more members of the household under 5 years of age	Dummy variable: 1 = yes, 0 = no
	At least one or more members of the household 65 or older	Dummy variable: 1 = yes, 0 = no
Gender	Gender of the household head	Dummy variable: 1 = male, 2 = female
Health insurance coverage	At least one member of the household is covered by health insurance	Dummy variable: 1 = yes, 0 = no
	Head of the household is covered by health insurance	Dummy variable: 1 = yes, 0 = no
Employment*	At least one employed member in the household	Dummy variable: 1 = yes, 0 = no
	At least one unemployed member in the household	Dummy variable: 1 = yes, 0 = no
	At least one inactive (not defined as employed or unemployed) member in the household	Dummy variable: 1 = yes, 0 = no
	At least one disabled member in the household	Dummy variable: 1 = yes, 0 = no
	Employment status of the head of the household	Dummy variable: 1 = yes, 0 = no
Payment for inpatient care	Has any member of the household paid for inpatient care in the past 12 months?	Dummy variable: 1 = yes, 0 = no
Settlement	Is household in urban or rural area?	Dummy variable: 1 = rural, 0 = urban
Expenditure quintiles in PPP-adjusted Euros	Households ranked in five equal groups according to annual HCE per adult equivalent (excluding durable goods and rent) in increasing order to the expenditure cutoff point in each quintile	Ordinal: Lowest = 14.69–222.32
		II = 222.48–291.58
		III = 291.74–364.73 IV = 364.83–474.86
		Highest = 474.95–1885.90
Health care utilization	At least one member of the household was hospitalized during the previous 12 months	Dummy variable: 1 = yes, 0 = no

* HBS activity variable with the longest duration during the previous 12 months [32]

To identify the factors associated with catastrophic health expenditures (household characteristics), we used bivariate statistics (Chi square test) to identify potential covariables to be tested in the multivariable model. We used a threshold of p < 0.1 to determine the statistical significance of the potential pairs of variables in the multivariable analyses so that we would not miss any potentially relevant variables for catastrophic health expenditures.

The Pearson’s R correlation coefficient was used to identify pairs of variables that were correlated. Correlations of r > 0.5 are considered collinear. We tested for multicollinearity and estimated variance inflation factors (VIF), tolerance, R squared and condition index by applying common rule-of-thumb cutoff points [34]. In cases where two or more pairs of variables were found to be collinear, the variable that was most strongly associated with catastrophic health expenditures and reasonably conceptualized in relation to it was retained. Variables were included in the model if p < 0.05 and removed from the model if p > 0.1, except those that we considered important to present considering the context. The backward stepwise method for model building was used to identify statistically significant variables that determined catastrophic health expenditures at the 10% household consumption cutoff level. We conducted nested likelihood ratio tests for expenditure quintiles and having one or more household member who is less than 5 years of age, 65 years or above, or disabled. In all tests, the differences were found to be statistically significant.

We used STATA release 14 to analyze the data in this study [35]. We used Microsoft Office Excel for Mac release 2011 for the direct assessment and presentation of progressivity of the share of OOP health payments in HCE.

## Results

In Table 2, descriptive analyses of the household characteristics are presented. The average household size in Kosovo was estimated as 5.49, with approximately half of households (55.16%) having at least one member equal to or less than 5 years old or equal to or more than 65 years old.

**Table 2:** Description of Kosovo household characteristics, 2014, N = 2375

Variable name	Mean (SD) or %
Mean annual HCE per adult equivalent (in PPP-adjusted Euros)	361.21 (183.37)
Mean household size	5.49 (2.65)
Mean age of the household head	53.08 (13.33)
Households with at least one member 5 years old or younger (%)	27.41
Households with at least one member 65 years old or older (%)	35.63
Education of household head, primary or lower (%)	37.07
Households with male head (%)	91.94
Head of the household covered by health insurance (%)	7.68
Households with at least one member covered by health insurance (%)	8.33
Households with employed head (%)	57.95
Households with at least one member employed (%)	78.75
Households with a disabled member during the previous 12 months (%)	5.24
Households with at least one member having been hospitalized during the previous 12 months (%)	17.71

We found that almost all households were headed by a male member (91.94%), and 37.07% of household heads had primary or lower-level education. Only 7.68% of household heads were covered by a health insurance scheme, whereas 8.33% of households had at least one member covered by a health insurance scheme as insuree or as a dependent of an insured household member.

The mean annual HCE per adult equivalent was estimated to be 335.71 PPP-adjusted Euros. Heads of households were employed in 57.95% of the households, whereas 78.75% of the households had at least one member who was employed in the previous 20 months.

Approximately 5.24% of the households had at least one disabled member, and 17.71% of the households had at least one member hospitalized during the previous 12 months.

Table 3 shows that households that incurred OOP health payments (80.76%) paid on average 128.01 PPP-adjusted Euros. The mean OOP health payment, including households with no OOP spending on healthcare, was 100.88 PPP-adjusted Euros per household. Among households that incurred OOP health payments, the highest average amounts were paid for treatment abroad (797.83 PPP-adjusted Euros) and hospital services (133.56 PPP-adjusted Euros), followed by dental services (59.17 PPP-adjusted Euros), diagnostic services (57.58 PPP-adjusted Euros) and outpatient services (43.95 PPP-adjusted Euros). Treatment abroad totaled 2.1 million PPP-adjusted Euros.

**Table 3:** Household annual out-of-pocket (OOP) health payments (PPP-adjusted Euros) by area of expenditure, 2014 HBS

OOP health payments	Number of households that incurred OOP (N)	Households that incurred OOP (%)	Mean OOP per households that incurred OOP	Mean OOP per all households (n = 2375)	Mean OOP per household member who participated in survey
Medicine, pharmaceutical products and medical devices	1891	79.62	94.82	73.74	13.43
Outpatient services	328	13.81	43.95	5.79	1.05
Dental services	136	5.73	59.17	3.08	0.56
Diagnostic services	342	14.40	57.58	7.49	1.36
Hospital services	80	3.37	133.56	3.85	0.70
Treatment abroad	23	1.05	797.83	6.59	1.20
Other medical services	37	0.02	24.22	0.32	0.06
OOP for all health care services combined	1918	80.76	128.01	100.88	18.37

With 13.43 PPP-adjusted Euros per household member, most of the OOP health payments were incurred for medicines, pharmaceutical products and medical devices (79.62%), followed by diagnostic services (14.40%), outpatient services (13.81%), and dental services (5.73%).

**Fig. 1:**
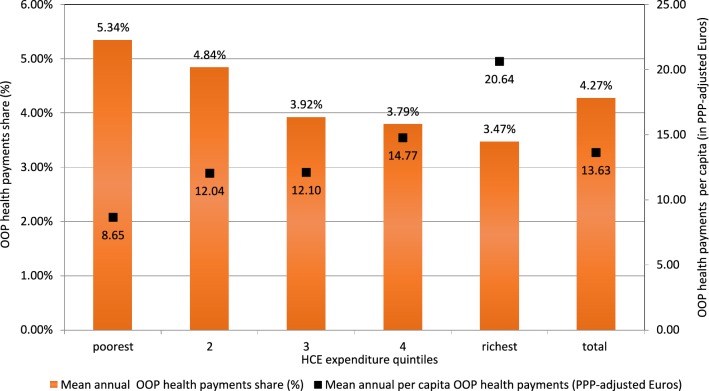
Average annual out-of-pocket health payments shares and per capita monetary values (in PPP-adjusted Euros) across household consumption expenditure quintiles, 2014

Although households with higher consumption expenditures spent more on OOP health payments than households with lower consumption expenditures, the latter spent a greater share of their consumption expenditures on healthcare. OOP health payments per capita accounted for 5.34% (on average, 8.65 PPP-adjusted Euros) in the lowest consumption expenditure quintile and 3.47% (20.64 PPP-adjusted Euros) in the highest consumption expenditure quintile. On average, households in Kosovo spent 4.27% of their budget on healthcare, as depicted in Fig. 1.

Table 4 provides an overview of catastrophic health expenditures by measuring their incidence and intensity at different thresholds of health payments as shares of consumption expenditures (non-food expenditures). As the threshold z for the share of OOP health payments among HCE increased from 5 to 25%, the incidence of catastrophic health expenditures decreased from 29.63 to 1.69%, and the overshot dropped from 1.92 to only 0.17%. The mean positive overshot (average percentage of OOP health payments among only those households that exceed the threshold z) did not decline as the threshold increased. Those who paid more than 10% of their HCE on healthcare on average spent 17.21% (10% + 7.21%).

**Table 4:** Incidence and intensity of out-of-pocket (OOP) health payments for healthcare (%), 2014

Catastrophic health expenditure measures	Threshold z budget share				
OOP health payments as share of HCE	5%	10%	15%	25%	40%
Head count (H)	29.63	13.01	5.87	1.69	–
Standard error for H	0.99	0.71	0.50	0.26	
Overshot (O)	1.92	0.94	0.48	0.17	–
Standard error for O	0.10	0.08	0.06	0.04	
Mean positive overshot (MPO)	6.50	7.21	8.09	10.30	–

As share of non-food consumption expenditures					
Head count (H)	–	–	30.24	16.01	5.34
Standard error for H			1.00	0.79	0.48
Overshot (O)	–	–	4.47	2.25	0.75
Standard error for O			0.22	0.16	0.09
Mean positive overshot (MPO)	–	–	14.78	14.05	14.04

The overshot (average percentage of OOP health payments that exceed the threshold z across the entire sample) indicates a low average percentage of OOP health payments that exceeded any threshold as a share of both household consumption and non-food consumption expenditures, whereas the mean positive overshot (OOP health payments’ average percentage only among households that exceed the z threshold) was higher. Those who paid more than 25% of their household budget respectively non-food consumption expenditures spent on healthcare on average spent 35.30% (25% + 10.30%), respectively 39.05% (25% + 14.05).

The share of OOP payments for healthcare was always higher within non-food consumption than within HCE. For example, Table 4 also indicates that for 16.01% of the households, health spending was at least one-quarter of non-food expenditures, but health spending was at least one-quarter of HCE for 1.69% of households.

We estimated an annual poverty line of 217.09 PPP-adjusted Euros per adult equivalent. Table 5 shows that based on the 2014 HBS, 20.70% of Kosovo’s population lived below the poverty line.

**Table 5:** Poverty head count (%) and poverty gap (PPP-adjusted Euros) due to out-of-pocket health payments

Poverty head count	Total	Urban	Rural
Pre-payment head count	20.70	20.83	20.62
Post-payment head count	22.21	21.60	22.61
Percentage point change (absolute)	1.50	0.77	1.99
Percentage change (relative)	7.26	3.68	9.66

Poverty gap	Total	Urban	Rural
Pre-payment poverty gap	10.33	10.33	10.33
Post-payment poverty gap	11.44	11.36	11.50
Percentage point change (absolute)	3.40	3.14	3.57
Percentage change (relative)	10.76	9.95	11.30

Due to rounding, some percentages may not correspond exactly with the separate figures

Table 5 also indicates that OOP health payments increased the poverty head count by 22.21% (relative percentage change). In 2014, there was a 7.26% increase in the head count ratio due to OOP health payments. After making OOP health payments, more people became poor in rural compared to urban areas. The Chi square test failed to detect a difference between urban and rural areas in the proportional increase in poverty due to OOP health payments.

The pre- and post-payment poverty gap indicates the extent to which OOP health payments further impoverish the population. In 2014, OOP health payments increased the poverty gap by almost 11.30%. This increase was slightly higher in rural than in urban areas.

Table 6 presents the estimated odds ratios from a multivariable logistic regression model for a catastrophic health expenditures threshold at the 10% level of HCE. Bivariate statistics (Chi square) did not identify the residential area (urban/rural) as a potential co-variable to be tested in the multivariable model.

**Table 6:** Determinants of catastrophic health expenditures in Kosovo, 2014

Variable	Odds ratio	Standard error	T	p > |t|	95% confidence interval	
HCE expenditure quintile^a^						
Quintile 1 vs 5	2.466	0.487	4.57	0.001	1.675	3.633
Quintile 2 vs 5	1.661	0.328	2.57	0.010	1.128	2.445
Quintile 3 vs 5	0.922	0.193	− 0.39	0.696	0.612	1.388
Quintile 4 vs 5	0.897	0.186	− 0.52	0.601	0.597	1.347
Age of the household head	1.011	0.005	2.03	0.042	1.000	1.021
Household size	0.807	0.029	− 6.06	0.001	0.753	0.865
Member under 5 years old in the household	1.133	0.178	0.80	0.425	0.833	1.541
Member over 65 years old in the household	1.971	0.283	4.73	0.001	1.488	2.612
At least one employed member in the household	0.981	0.155	− 0.12	0.904	0.719	1.338
Disabled member in the household in previous 12 months	2.819	0.599	4.88	0.001	1.859	4.276
Insurance coverage of household head	0.275	0.092	− 3.87	0.001	0.143	0.528
Education level of household head	1.113	0.148	0.81	0.419	0.858	1.445
Constant	0.134	0.060	− 4.5	0.001	0.056	0.322

OOP health payments divided by HCE ≥ 10%

^a^Quintile 1 has the lowest consumption expenditures and quintile 5 the highest

The empirical results from the logistic regression analysis shown in Table 6 reveal that there was a lower probability of catastrophic health expenditures when the household head has insurance coverage. Economic status as measured by consumption expenditure quintiles was significantly associated with catastrophic health expenditures. Compared to the highest income quintile, progressively lower income quintiles had higher odds of catastrophic expenditures. The regression analysis found that the age of the household head, insurance coverage, household size, belonging to the two poorest consumption expenditure quintiles, and having disabled and aged household members were significant predictors of the probability of experiencing catastrophic health expenditures.

## Discussion

We found that during 2014, 80.76% of the households in the sample incurred OOP health payments. OOP payments represented approximately one-third of the total health care expenditures and showed a slightly regressive socioeconomic gradient and an unchanged impoverishment effect and poverty gap compared to 2011 [19, 36].

In 2014, OOP health payments constituted 33.30% of total health spending [36]. This share remains high when compared to the upper limit of 15–20% recommended by the WHO and the average of the European Union member states (23.1%) but is comparable to that of neighboring Macedonia at 31.1% and Serbia at 37.9% [19, 37]. Evidence from the literature shows that when OOP health payments are less than 20% of total health expenditures, the incidence of catastrophic health expenditures is usually negligible [38]. This finding indicates that Kosovo does not provide sufficient financial risk protection, which is generally most relevant for the population segment with the fewest resources. In this study, OOP health payments represent 4.27% of HCE, which is higher than the average for OECD countries (3.2%) [39].

Most of the OOP health payments incurred were for medicines, pharmaceutical products and medical devices. These costs may also be a result of a limited essential drug list (positive list) and a lack of pricing regulation in the private market, which accounts for approximately 85% of the total pharmaceutical market [40]. Moreover, these costs may be attributed to physician-induced demand in the absence of common treatment guidelines and a robust information system. Such a system would monitor prescriptions and the dispensing of them at pharmacies [19]. To this end, some essential regulatory measures are required, such as definitions of the most common treatment guidelines, reference pricing for drugs, revision of the essential drugs list, development of a pharmaceutical information system module, and regulations that can enable contracting pharmacies to dispense outpatient drugs. These are some of the immediate measures that would address all of the above-mentioned factors and prevent unnecessary increases in OOP health payments [41].

To the best of our knowledge, our estimates of OOP health payments for treatment abroad are the first to be performed at the national level for Kosovo. We found that treatment abroad was sought at a low frequency but at high cost. Patients in Kosovo continue to obtain health services abroad, and we estimate the total cost at 2.1 million PPP-adjusted Euros, which is a more plausible and much lower figure than the approximated 26.2 million PPP-adjusted Euros previously reported [42]. In the period between 2006 and 2009, the government of Kosovo spent on average approximately 2059.73 PPP-adjusted Euros per case sent for treatment abroad [43]. The reasons for seeking treatment abroad and the types of healthcare services used abroad should be examined more closely. In the future, reforms should aim to reduce the number of patients seeking treatment abroad by offering such services domestically and to ensure that these services remain accessible to households regardless of their economic status [43].

This study measured the level of financial risk protection in Kosovo through two commonly applied statistical parameters, i.e., catastrophic health expenditures and impoverishment, analyzed and identified potential causes and recommended policies with concrete actions. Considering the different methodologies and variable definitions, the proportion of households facing catastrophic health expenditures in Kosovo in 2014 was similar to that in 2009 but higher than that in 2011 [13, 19, 44]. The proportion estimated in 2014 is comparable to the latest available data for Albania and higher than that for Serbia [45, 46]. Kosovo has all three of the factors that mainly give rise to catastrophic health expenditures: (i) health services requiring OOP health payments; (ii) low household capacity to pay; and (iii) a lack of pre-payment mechanisms for risk pooling [33]. To this end, Kosovo’s government is considering increasing its total health spending by introducing mandatory health insurance contributions in addition to the country’s already existing mainly tax-based public health spending. The healthcare system reforms in Kosovo aim to improve the population’s health status through universal access to high-quality basic health care services without financial hardship. This will primarily require a targeting mechanism to identify households that are more likely to be confronted by catastrophic health expenditures. Our study indicates that a higher age of the household head, belonging to the two poorest quintiles and having disabled and aged members of the household significantly increase the likelihood of being confronted with catastrophic health expenditures.

In 2014, impoverishment due to OOP health payments and the poverty gap remained stable compared to 2011 [19]. Households in higher consumption expenditure quintiles spent more on OOP health payments; however, households in lower quintiles spent a higher share of their consumption expenditures on healthcare. Compared to 2011, these data indicate a tendency for OOP health payments to increase inequality [19]. To explain this tendency, in line with the findings of another study, we consider that two main factors negatively affect the equality of the distribution of OOP health payments in Kosovo: (i) income inequality and (ii) the inability of the healthcare system to provide care without regard to a household’s ability to pay [47]. The Gini coefficient (with a value of 0 indicating total equality, and a value of 100% indicating total inequality of the income distribution among a country’s residents) for Kosovo is estimated at 27.6%, which is similar to that for developed countries [32]. However, Kosovo has limited welfare benefits and available social capital. Therefore, targeting the poor through the upcoming compulsory health insurance scheme should be a priority.

The concept of catastrophic health expenditures is considered a risky choice to invest in one’s health, as are all types of investments [48]. However, the human rights approach to health considers investing in health a necessity, even when there is no return on “future labor income.”

### Limitations

Limitations of the study are related to survey instrument design and the methodology used. Compared to specialized health surveys, the HBS is known to underestimate OOP health payment data, since it collects data on all types of household expenditures. The HBS is also known for limitations related to non-sampling errors; however, it remains the most comprehensive and reliable data source on household expenditures [49]. Comparing the pre- and post-payment head count is a rough indicator of the impoverishment effect due to OOP health payments, since we cannot be certain that an incurred health expenditure was completely nondiscretionary (necessary), that resources remain fixed and that households do not use their savings, borrow or sell their assets [6]. Uncorrected estimates for financial coping mechanisms may have led to an overestimation of impoverishing effect of OOP health payments [50]. Our study does not identify those who forgo care, since they cannot afford healthcare payments and therefore do not incur OPP health payments. Finally, because the study focuses on consumption expenditures, it could not measure the impact of opportunity costs, such as income losses due to illness, socioeconomic shocks or deaths.

## Conclusions

Monitoring financial risk protection through HBS data remains an important mechanism in low and middle-income countries. This study indicates that monitoring financial risk protection should move beyond the most commonly applied statistical parameters such as catastrophic health expenditures and impoverishment and include other parameters from national health accounts when they are available, such as total healthcare spending. This parameter is particularly important for low and middle-income countries, in which the share of public spending is commonly low. The relevant stakeholders can use this fact to advocate for an increased public share of healthcare spending. Holding countries accountable to improve the level of financial risk protection cannot be seen separately from ensuring that essential health services include risk protection policies. Such accountability would require reliable data collection and analytical capacity as part of regular monitoring of health system performance as countries ideally move toward UHC. To this end, in the absence of reliable data from healthcare institutions, the HBS survey deserves special attention as a comprehensive and disaggregated information source on not only the level of financial protection but also that of healthcare coverage.

Further research should focus on analyzing the degree of forgone care and the magnitude of other resources used to cover OOP health payments to reflect how well the catastrophic health expenditures and impoverishment in Kosovo have been approximated. Moreover, the implications of OOP health payments for equitable financing should be monitored closely.

We conclude that for Kosovo, OOP health payments still represent a high share of total health expenditures. The healthcare system in Kosovo does not provide well-targeted financial risk protection for the poorest segment of the population. The increasing share of HCE on health across the lower expenditure quintiles highlights the need to understand the implications of such expenditures for equitable financing. Closely monitoring the level of financial risk protection and understanding the change of measures to assess the equity of OOP health payments remain important to develop appropriate and evidence-based recommendations for policy makers. Targeting the poor, revisiting pharmaceutical policies and strengthening the institutional referral system for treatment should be considered priorities to improve the performance of the healthcare system in Kosovo.

## Abbreviations

CPI: consumer price index
HCE: household consumption expenditures
HBS: Household Budget Survey
KAS: Kosovo Agency of Statistics
PPP: purchasing power parity
OECD: Organization for Economic Co-operation and Development
OOP: out-of-pocket
UHC: universal health coverage
WB: World Bank
WHO: World Health Organization

## References

[1] United Nations General Assembly. Sixty-seventh session. Agenda item 123, Global health and foreign policy.In: Non-communicable Diseases Alliance; 2012. https://ncdalliance.org/sites/default/files/resource_files/Global%20Health%20and%20Foreign%20Policy%20resolution%202012_67th%20GA.pdf. Accessed 1 July 2018.

[2] World Health Organization and the International Bank for Reconstruction and Development, the World Bank. Tracking universal health coverage: 2017 global monitoring report: executive summary. In. Institutional Repository for Information Sharing; 2017. http://apps.who.int/iris/handle/10665/260522. Accessed 1 July 2018.

[3] World Health Organization. Universal health coverage: from technical report to global movement. In: World Health Organization Regional Office for Europe, health topics, health system, health financing; 2012. http://www.euro.who.int/en/health-topics/Health-systems/health-systems-financing/news/news/2012/11/universal-health-coverage-from-technical-report-to-global-movement. Accessed 1 July 2018.

[4] Kutzin J (2013). Health financing for universal coverage and health system performance: concepts and implications for policy. Bull World Health Organ.

[5] Li Y, Wu Q, Legge D, Hao Y, Gao L, Ning N, Wan G (2012). Factors affecting catastrophic health expenditure and impoverishment from medical expenses in China: policy implications of universal health insurance. Bull World Health Organ.

[6] Wagstaff A, Flores G, Smitz MF, Hsu J, Chepynoga K, Eozenou P (2017). Progress on impoverishing health spending in 122 countries: a retrospective observational study. Lancet Global Health.

[7] Chuma J, Maina T (2012). Catastrophic health care spending and impoverishment in Kenya. BMC Health Serv Res.

[8] Knaul FM, Arreola-Ornelas H, Méndez-Carniado O, Bryson-Cahn C, Barofsky J, Maguire R, Miranda M, Sesma S (2006). Evidence is good for your health system: policy reform to remedy catastrophic and impoverishing health spending in Mexico. Lancet.

[9] Yardim N, Yardim MS, Cilingiroglu N (2010). Catastrophic health expenditure and impoverishment in Turkey. Health Policy.

[10] Özgen Narci H, Şahin I, Yıldırım HH (2015). Financial catastrophe and poverty impacts of out-of-pocket health payments in Turkey. Eur J Health Econ.

[11] Lu C, Chin B, Li G, Murray CJL (2009). Limitations of methods for measuring out-of-pocket and catastrophic private health expenditures. Bull World Health Organ.

[12] Xu K, Evans DB, Kawabata K, Zeramdini R, Klavus J, Murray CJ (2003). Household catastrophic health expenditure: a multicountry analysis. Lancet.

[13] Bredenkamp C, Mendola M, Gragnolati M (2011). Catastrophic and impoverishing effects of health expenditure: new evidence from the Western Balkans. Health Policy Plan.

[14] van Doorslaer E, O’Donnell O, Rannan-Eliya RP, Somanathan A, Adhikari SR, Pande BR, Garg CC (2006). Effect of payments for health care on poverty estimates in 11 countries in Asia: an analysis of household survey data. Lancet.

[15] Leive A, Xu K (2008). Coping with out-of-pocket health payments: empirical evidence from 15 African countries. Bull World Health Organ.

[16] Knaul FM, Wong R, Arreola-Ornelas H, Méndez O (2011). Household catastrophic health expenditures: a comparative analysis of twelve Latin American and Caribbean Countries. Salud Publica Mex.

[17] Xu K, Evans DB, Carrin G, Aguilar-Rivera AM, Musgrove P, Evans T (2007). Protecting households from catastrophic health spending. Health Aff (Millwood).

[18] World Bank. Kosovo healthcare financing reform study. In: Open knowledge repository. World Bank; 2008. https://openknowledge.worldbank.org/handle/10986/8121. Accessed 1 July 2018.

[19] World Bank. Kosovo public finance review, fiscal policies for a young nation. In: Documents and reports. World Bank; 2014. http://documents.worldbank.org/curated/en/2014/06/20345957/kosovo-public-finance-review-fiscal-policies-young-nation. Accessed 1 July 2018.

[20] European Commission: EU Health Policy; 2018. https://ec.europa.eu/health/policies/overview_en. Accessed 1 July 2018.

[21] Shuey DA, Qosaj FA, Schouten EJ, Zwi AB (2003). Planning for health sector reform in post-conflict situations: Kosovo 1999–2000. Health Policy.

[22] Assembly of the Republic of Kosovo. Law No. 04/L-125 on Health. In: Laws by name. Assembly of Republic of Kosovo; 2012. http://www.kuvendikosoves.org/common/docs/ligjet/Law%20on%20Health.pdf. Accessed 1 July 2018.

[23] Assembly of Republic of Kosovo. Law No. 04/L-249 on health insurance. In: Laws by name. Assembly of Republic of Kosovo; 2014. http://www.kuvendikosoves.org/common/docs/ligjet/04-L-249%20a.pdf. Accessed 1 July 2018.

[24] Joint Learning Network for universal health coverage: universal access to care in Kosovo; 2014. http://www.jointlearningnetwork.org/countries/kosovo. Accessed 1 July 2018.

[25] Eurostat (2003). Household budget surveys in the EU. Methodology and recommendations for harmonisation.

[26] Kosovo Agency Statistics. Results of household budget survey 2014. In: Serie 5 social statistics. Kosovo Agency of Statistics; 2015. http://ask.rks-gov.net/media/1503/results-of-household-budget-survey-2014.pdf. Accessed 1 July 2018.

[27] Eurostat. In: Classification of individual consumption by purpose (COICOP) five digit structure and explanatory notes; 2013. http://www.dst.dk/ext/pris/coicop–pdf. Accessed 1 July 2018.

[28] Riinvest. Publications. In: A business perspective of informality in Kosovo. Friedrich-Ebert-Stiftung Kosovo; 2013. http://www.fesprishtina.org/wb/media/Publications/2013/BUSINESS_INFORMALITY__ENG_FINAL.pdf. Accessed 1 July 2018.

[29] Douarin E, Litchfield J, Sabates-Wheeler R. Poverty, livelihoods and war legacies: the case of post-war rural Kosovo. In: Institute of Development Studies, working Papers; 2011. https://www.ids.ac.uk/files/dmfile/Wp380.pdf. Accessed 1 July 2018.

[30] Berisha IT, Asllani I, Gashi R (2015). Economic statistics: consumer price index 2014.

[31] World Bank. International comparison programe (ICP). Data bank; 2005. http://databank.worldbank.org/data/reports.aspx?source=international-comparison-program-(icp)-2005.

[32] Simler K, Miyata S, Gyulnazaryan Y, Bidani B, Bellaqa B, Deliu E, Haqifi B (2011). Consumption poverty in the Republic of Kosovo in 2009.

[33] Xu K, Evans D, Carrin G, Aguilar-Rivera AM. Designing health financing systems to reduce catastrophic health expenditure. In: Health financing for universal coverage. Document center; 2005. http://www.who.int/health_financing/documents/cov-pb_e_05_2-cata_sys/en/. Accessed 1 July 2018.

[34] Williams R: Multicollinearity; 2015. https://www3.nd.edu/~rwilliam/stats2/l11.pdf. Accessed 1 July 2018.

[35] StataCorp (2015). Stata statistical software: release 14.

[36] National Institute of Public Health Kosovo (2015). Estimation of total health spending in Kosovo 2014.

[37] World Health Organization. European health for all database. World Health Organization; 2014. http://data.euro.who.int/hfadb/param.php. Accessed 1 Nov 2015.

[38] World Health Organization (2010). World health report 2010, health systems financing: the path to universal coverage.

[39] OECD: Health at a Glance 2011: OECD Indicators. OECD; 2011. https://www.oecd.org/els/health-systems/49105858.pdf. Accessed 1 July 2018.

[40] Imasheva A, Seiter A. The pharmaceutical sector of the western Balkan Countries. In: Documents and reports. World Bank; 2008. http://documents.worldbank.org/curated/en/2008/02/9071523/pharmaceutical-sector-western-balkan-countries. Accessed 1 July2018.

[41] van den Berg B, Spreeuwenberg PMM, Groenewegen PP, van Dijk CE, Verheij RA, de Bakker DH (2012). Moral hazard and supplier-induced demand: empirical evidence in general practice. Health Econ.

[42] Bytyqi S. Investigation into public health in Kosovo. Balkan Policy Institute; 2012.

[43] World Bank. Kosovo public expenditure review. In: Documents and Reports. World Bank; 2010. http://documents.worldbank.org/curated/en/2010/06/12507653/kosovo-public-expenditure-review. Accessed 1 July 2018.

[44] Goryakin Y, Suhrcke M (2014). The prevalence and determinants of catastrophic health expenditures attributable to non-communicable diseases in low- and middle-income countries: a methodological commentary. Int J Equity Health.

[45] Tomini SM, Packard TG, Tomini F (2012). Catastrophic and impoverishing effects of out-of-pocket payments for health care in Albania: evidence from Albania living standards measurement surveys 2002, 2005 and 2008. Health Policy Plan.

[46] Arsenijevic J, Pavlova M, Groota W (2013). Measuring the catastrophic and impoverishing effect of household health care spending in Serbia. Soc Sci Med.

[47] Markova N. How Does the introduction of health insurance change the equity of health care provision in Bulgaria. International Monetary Fund, working paper; 2006. https://www.imf.org/external/pubs/ft/wp/2006/wp06285.pdf. Accessed 1 July 2016.

[48] Peter Z (2016). ‘Catastrophic’ healthcare expenditure: critique of a problematic concept and a proposal. Eur J Health Econ.

[49] Ravi P. National Health accounts estimation methods: household out-of-pocket spending in private expenditure. In: OECD; 2008. http://www.oecd.org/els/health-systems/37808429.pdf. Accessed 1 July 2018.

[50] Peter B, Rajeev A, Laveesh B (2010). The impoverishing effect of healthcare payments in india: new methodology and findings. Econ Polit Weekly.

